# Concerns About Replicability Across Two Crises in Social Psychology

**DOI:** 10.5334/irsp.1036

**Published:** 2025-05-12

**Authors:** Daniël Lakens

**Affiliations:** 1 Eindhoven University of Technology, The Netherlands

**Keywords:** replication, methodology, history of psychology, ethics

## Abstract

Twice in the history of social psychology has there been a crisis of confidence. The first started in the 1960s and lasted until the end of the 1970s, and the second crisis dominated the 2010s. Drawing on extensive quotes from articles published during both crises, I examine the similarities and differences between these psychological crises. In this first of two articles, I focus on how researchers discussed fundamental concerns about the replicability of findings across the two crises. I reflect on five possible reasons why concerns about failed replications received more attention during the second crisis, the continuing lack of incentives to perform replication studies, and the importance of large-scale research projects to instigate change.

Everything that happens once can never happen again. But everything that happens twice will surely happen a third time.

*Paulo Coelho, The Alchemist*.

Twice in the history of social psychology has there been a crisis of confidence. The first started in the 1960s and lasted until the end of the 1970s. Bakan (1967, p. xii) states, ‘I believe there is a crisis in research in psychology,’ and Elms writes:

‘Many social psychologists appear to have lost not only their enthusiasm but also their sense of direction and their faith in the discipline’s future. Whether they are experiencing an identity crisis, a paradigmatic crisis, or a crisis of confidence, most seem agreed that a crisis is at hand’.

The second crisis dominated the 2010s, and almost forty years after the first crisis, Pashler and Wagenmakers (2012, p. 528) wrote, ‘Is there currently a crisis of confidence in psychological science reflecting an unprecedented level of doubt among practitioners about the reliability of research findings in the field? It would certainly appear that there is’. Although there are some differences between these two crises, there are also striking similarities. Many of the problems that were raised in the first crisis have resurfaced in the second crisis, suggesting that the first crisis was largely unsuccessful in resolving fundamental problems in the field. Looking back on the major developments in the history of social psychology, Jones remarks, ‘The crisis of social psychology has begun to take its place as a minor perturbation in the long history of the social science. The intellectual moment of the field has not been radically affected by crisis proclamations’. Will the second crisis lead to more radical changes, or will it eventually also be largely forgotten? Which problems were successfully addressed in the first crisis, which challenges have remained unaddressed, and why? And how can we learn from our history to prevent the same fundamental problems from resurfacing in a third crisis in the future? In this manuscript, I will review discussions concerning the replicability of findings across these two crises in the history of psychology, and in a second manuscript, I will review the literature discussing the state of theories in psychology, the practical relevance of psychological research, the generalizability of findings, and methodological problems.

One goal of these manuscripts is to introduce a readership that might not be aware of the similarities between these two crises to the thoughts and discussions of a previous generation of academics. I am not an historian of psychology, and these manuscripts do not aim to provide a historical analysis of both crises, as these can be found elsewhere (Malich & Munafò, 2022; Morawski, 2015; Mülberger, 2012; Mülberger, 2022), but I believe that it is important to educate a younger generation about the similarities and differences between two crises in psychology. Where relevant, I will add my personal experiences as a researcher who has worked on some of the challenges that were discussed during these crises, with the hope that reflections from an insider’s perspective will provide useful information for future historians of psychology. I have chosen to focus on five topics that were discussed more or less extensively during both crises: replication, theory, relevance, generalizability, and methodology. The topics of research ethics and measurement validity are primarily criticized in the first and the second crisis, respectively, and are discussed in the introduction (ethics) and discussion (measurement and validity). Researchers have pointed out concerns regarding all these aspects of the scientific process, as well as other perceived problems, throughout the history of psychology. But the intensity with which these topics have been discussed increased substantially during the two crises, and during a crisis researchers typically reflect on how multiple of these problems are interrelated instead of focusing on a single issue. The focus on these seven topics is necessarily selective. The choice for these topics is based both on the attention these topics have received in the literature during both crises, as a personal evaluation of their importance.

## Is there a crisis?

Many researchers have used the term ‘crisis’ over the last century to discuss what they considered to be fundamental challenges to their field (Sturm & Mülberger, 2012). Both the subjective experience of a crisis and the empirical support for the presence of a crisis have been points of contention during each crisis. Both crises are strongly associated with research in social psychology, even though most of the problems that have been identified are equally relevant to other subfields in psychology or even other scientific disciplines, such as sociology (Boutilier et al., 1980; Gouldner, 1970). Furthermore, not all of the researchers who have published on topics related to the two crises work in (social) psychology. Finally, many social psychologists feel the criticisms leveled against their field only apply to some research lines, while they believe other research lines are in much better shape.

Despite these caveats, social psychologists have been especially willing to question practices in their own field. Deutsch (1976, p. 134) already remarked, ‘The crisis in social psychology is not new; we are in a perpetual crisis’. And yet, during the two crises the concerns among scientists were especially widespread. There was a continuing discussion in top journals in the field, often based on contributions by leaders in the field, which lasted for more than a decade. Reflecting on the first crisis, Mills (1979) writes:

‘We, and social psychology generally, have undergone a crisis, not simply of confidence, as Elms suggests, but, more profoundly, of paradigm, of our general form of thought. It was as though the life-giving substance in the air we breathed became insufficient; some gasped and suffocated. It was as though we were fish out of water; some flapped around on dry ground. Although painful, the crisis is serendipitous to the extent that the early translucent form of thought becomes, through its inadequacy, more apparent, both in its productive and counterproductive features and, consequently, more accessible for appreciation and redesign’.

Nederhof and Zwier (1983) surveyed researchers in psychology at the end of the first crisis about their subjective perception and found that 87.6% of active researchers agreed with the statement that ‘During the last decade, social psychologists have shown much concern over the state of their discipline’, and 34.1% of the scientists still agreed with the item ‘There is a crisis in social psychology’ at the end of the first crisis. During the second crisis, a survey across scientific disciplines similarly revealed that many researchers subjectively experienced a crisis of confidence (Baker, 2016).

Beyond these surveys, it is notable that highly successful social psychologists admitted to personally believing the field was in a crisis. In 1970, Berkowitz wrote in personal correspondence to M. B. Smith (1972):

‘At any rate, it seems to me… that social psychology is now in a “crisis stage,” in the sense that Kuhn used this term in his book The Structure of Scientific Revolutions. We seem to be somewhat at a loss for important problems to investigate and models to employ in our research and theory. It is certainly time to take stock, to see where we are and where we should go…’

It should be noted that it is doubtful that any crisis in psychology meets the (arguably vague) requirements of a Kuhnian crisis (Sturm & Mülberger, 2012). Sherif (1977) writes:

‘Social psychology in this country is going through an ironic and unsettling state in its development. Ironic and unsettling, because on the surface it is thriving in a sky-rocketing boom of output in research and publication and, at the same time, the ratio of chaff that is piling up is enormous relative to the scanty yield in substance that will survive. This contradictory state of things is at the bottom of both the crisis and the unsettling malaise it arouses’.

Other psychologists agreed there were problems to address but believed the crisis narrative was overblown.

‘In this brief response, I want to suggest that the problem is more fundamental (and the solution less practically difficult) than has previously been considered in the burgeoning literature on the “crisis” of social psychology. While I agree with the critics that we are not sufficiently applied, theoretical, field oriented, or historical in our approach, and that some major rethinking is necessary, it also seems that we have made significant progress that can be the basis for real, satisfying success in the future’ (Aron, 1979).

Deutsch (1976, p. 134) writes, ‘Were I to engage in a polemic about theorizing in social psychology, my inclination would be to attack the “doom-criers”, those who assert that social psychology is in a “crisis which it must overcome if it is to survive”’. Others talk about ‘the alleged “crisis” in social psychology’ (Nederhof & Zwier, 1983) or the ‘so-called “crisis literature”’ (Senn, 1988). This use of quotation marks is also common among more critical voices during the second crisis, such as ‘the so-called “replication crisis” of the 2010s’ (Flis, 2019), with Stroebe and Strack (2014, p. 59) writing, ‘the alleged “crisis of replicability” is primarily due to an epistemological misunderstanding that emphasizes the phenomenon instead of its underlying mechanisms’. In this article, I will follow Goertzen’s (2008, p. 831) analysis of the term ‘crisis’, who writes:

‘I argue they mean there is a fundamental problem that is of serious importance. The adjectives from the previous sentence are significant here. The problem is fundamental — not tangential — to the discipline and its knowledge, and it is of serious — not passing — importance. I believe it is this combination of the problem being fundamental and serious that has led authors to refer to it as constituting a ‘crisis’ — despite the fact that a literal interpretation of that term is imprecise’.

Whether there is a fundamental problem of serious importance depends on two orthogonal underlying dimensions. The first dimension is the subjective experience of problems as being serious, and the second dimension is the extent to which the identified problems have a basis in reality. A field could, in principle, talk itself into a crisis when there are no problems or remain blissfully unconcerned about fundamental problems in scientific practice. During both crises, a substantial literature emerged that discussed concerns related to ethics, replication, theory, relevance, generalizability, methodology, and measurement. Collectively, this literature demonstrates the subjective experience of a problem, with a substantial number of articles providing either anecdotal evidence (mainly during the first crisis) or meta-scientific evidence (during the second crisis) of the presence of problematic research practices. Therefore, I consider the ‘crisis’ label appropriate.

## How did the crises start?

The first crisis started with concerns about research methods, the relevance of psychological research, and research ethics (Rosnow, 1981). Methodological criticism focused on the reliability of data collection from human participants due to demand and experimenter effects. The work by Rosenthal (whose 1966 book *Experimenter Effects in Behavioral Research* is still worth reading in full) was an important instigator, although I agree with the analysis of Barber and Silver (1968) that the evidence for experimenter bias presented by Rosenthal is often weak and, perhaps ironically, shows clear signs of inflated Type 1 error rates due to flexibility in how the data is analyzed. Concerns about the relevance of psychological research focused on limitations of lab-based experiments (Ring, 1967), further amplified by Gergen’s (1973) article ‘Social psychology as history,’ which challenged the idea that social psychology could produce generalizable knowledge.

Concerns about research ethics provided another important impetus to the first crisis. Questions were raised regarding the acceptability of deception and whether the negative consequences for subjects were balanced by the positive consequences of the knowledge that was gained. The obedience studies by Stanley Milgram specifically led to widespread discussions about research ethics (Baumrind, 1964; Milgram, 1964). While issues regarding research methods and relevance were not resolved in the first crisis and resurfaced during the second crisis, concerns about research ethics were addressed and led to a systemic change in research practices. Staff at the National Institutes of Health (NIH) in the US worked towards extending institutional review beyond medical research to the social sciences (Schrag, 2010). The American Psychological Association (APA), based on the belief that institutional review would be imposed on them if they did not organize it themselves, created a committee tasked with developing ethical principles (Stark, 2010). The scandal around the Tuskegee Syphilis Study broke in 1972. After consulting psychologists in the field, including those who had raised concerns in the psychological literature, the APA committee published the Ethical Principles in the Conduct of Research with Human Participants (1973).

Although institutional review boards receive their fair share of criticism (Peled-Raz et al., 2021), researchers are in general positive about the institutional review board process (Liddle & Brazelton, 1996; Malouff & Schutte, 2005; McNeill et al., 1992). Researchers who do not have access to ethical review boards indicate they would favor some form of a review board, share that they have often made mistakes in research ethics in the past when they had less experience, and believe they are not adequately trained in research ethics. Top-down regulations and administrative procedures will probably never become popular among academics, but they can be effective in changing behavior. In an ideal world where all scientists do the right thing, such administrative procedures would not be necessary. But we do not live in an ideal world, and no superior solution to prevent academics from violating ethical conduct has been developed. At the same time, researchers admit they do not always follow IRB procedures (Ashcraft & Krause, 2007), and there have certainly been side effects of implementing ethical review boards, including concerns about bureaucratization, time delays, overreach, and censorship (Schrag, 2010). Despite these challenges in implementing ethical review, the process has successfully resolved concerns raised during the first crisis about the ethical conduct of researchers.

The second crisis started in social psychology in 2011 with the publication of an article that provided empirical support for extrasensory perception (Bem, 2011), which coincided with the publication of a failed replication of a high-profile finding in the field (Doyen et al., 2012). These publications raised concerns about research practices that inflated the rate of false positives in the literature. Another impetus in the second crisis was a string of high-profile fraud cases, amplified by uncertainty about the difference between scientific fraud and research practices that inflate Type 1 error rates. As the concerns about replicability and methodological practices proved relevant for other disciplines as well, the second crisis rapidly spread beyond social psychology. The fact that history has repeated itself has not gone unnoticed among psychologists and fellow academics writing about the second crisis (Earp & Trafimow, 2015; Giner-Sorolla, 2012; Hales, 2016; Morawski, 2020; Pettigrew, 2018; Sharpe & Whelton, 2016), just as researchers writing during the first crisis recognized historical precedents (Lewin, 1977; Minton, 1984). Furthermore, even though the two crises are decades apart, some researchers have reflected on possible causes and solutions in both (e.g., Greenwald, 1976; Greenwald, 2012), and the aftermath of the first crisis was part of the training of psychologists who were senior during the second crisis.

## What is it a crisis of?

Even when many psychologists believed that the field was faced with fundamental problems, there is a noticeable pluralism during both the first and the second crisis about what researchers believe to be the causes of the crisis (Sherif, 1977). A crisis seems to provide authors with a useful vehicle to raise issues that they have been dissatisfied with for a long time. Similar themes emerge in both crises, albeit differing in prominence. Researchers debate 1) the replicability of findings, 2) the state of theories in their field, 3) the generalizability of research findings, 4) the practical relevance for society of the research the discipline produces, and 5) a concern about methodological practices. During both crises, researchers agree that many of these issues are caused by the incentive structure in academia, although the way incentives lead to this string of problems is complex. In this first manuscript, I will focus on similarities and differences between the two crises with respect to concerns about replicability. A second manuscript focuses on concerns about theorizing, relevance, generalizability, and methodology.

## A Replication Crisis

In an editorial in the Journal of Personality and Social Psychology, Greenwald (1976) writes:

‘There may be a crisis in personality and social psychology, associated with the difficulty often experienced by researchers in attempting to replicate published work. A precise statement of the magnitude of this problem cannot be made, since most failures to replicate do not receive public report’.

There are several similar statements in the literature, although there is a surprising lack of specificity in that none of the authors give examples of which findings have proven difficult to replicate. Festinger and Katz (1953, p. 64) state, ‘The history of social psychology illustrates the importance of the replication of findings in that many of its initial results have not been confirmed by later investigations’. McNemar already pointed out that due to publication bias in the published literature, ‘It follows that published results are more likely to involve false rejection of null hypotheses than indicated by the stated levels of significance. Perhaps this is one reason why replications by others so often fail to agree with so-called initial experiments’. A similar concern about the replicability of findings is expressed by Epstein (1980, p. 790): ‘Not only are experimental findings often difficult to replicate when there are the slightest alterations in conditions, but even attempts at exact replication frequently fail’.

When a finding fails to replicate, logically there are three possible reasons for a non-significant result. First, the original result could be a false positive, or Type 1 error. Second, the replication study yielded a false negative, or a Type 2 error. And third, the replication study might yield a non-significant result because there is an unknown moderator. Under some conditions, the effects can be replicated reliably, while under other conditions, the effect fails to emerge, but researchers do not yet understand what those conditions are (Schmidt, 2009). During the first replication crisis, the dominant belief was that replication failures should be attributed to an incomplete understanding of the conditions required to reliably observe an effect.

This idea is most clearly expressed by Sidman (1960, p. 63), who was also the first to extensively discuss the topic of replication in psychological research, when he writes:

‘Failure to replicate a finding, within or among species, is a result of incomplete understanding of the controlling variables. This positive approach, as contrasted to the negative attitude that failure to replicate must brand a process as nongeneral, is actually the only road to an adequate evaluation of generality’.

Even more explicitly, Sidman argues that pure chance (or a Type 1 error) should rarely be considered an explanation for a failed replication:

‘We are left, finally, with the basic problem of what is meant by “chance”. Are experimental observations ever the result of chance? To some experimenters, chance is simply a name for the combined effects of uncontrolled variables. If such variables are, in fact, controllable, then chance in this sense is simply an excuse for sloppy experimentation, and no further comment is required. If the uncontrolled variables are actually unknown, then chance is, as Boring has pointed out, a synonym for ignorance’.

This idea that failures to replicate emerge from a lack of understanding the required conditions to produce the effect requires a strong confidence in the reliability of earlier observations combined with the conviction that null effects do not exist in psychological research. Both these ideas continue to have proponents to this day.

If the main problem underlying the difficulty of replicating results is the presence of unknown moderators, then one possible reason for this state of affairs is that not sufficient information is provided in published articles to allow peers to perform a close replication study. For example, Pereboom writes, ‘Related to the above is the common difficulty of communicating all important details of a psychological experiment to one’s audience. […] Investigators attempting to replicate the work of others are painfully aware of these informational gaps’. In addition to more transparent communication about experimental conditions and procedures, researchers need to learn about when experiments replicate and when not. Replication studies should therefore be published in the scientific literature, which happens too rarely. Neher (1967, p. 262) concludes:

‘The general adoption of independent replication as a requirement for acceptance of findings in the behavioral sciences will require the efforts of investigators, readers, and publishing editors alike. It seems clear that such a policy is both long overdue and crucial to the development of a sound body of knowledge concerning human behavior’.

Lubin (1957) suggests that, where relevant, manuscripts that demonstrate the replicability of findings should receive a higher publication priority. Tullock (1959, p. 593) believes that ‘Journals should make space for brief reports of such repetitions, and foundations should undertake their support’. Lykken (1968, p. 159) writes, ‘Ideally, all experiments would be replicated before publication but this goal is impractical’. Loevinger (1968, p. 455) makes a similar point that ‘most studies should be replicated prior to publication. This recommendation is particularly pertinent in cases where the results are in the predicted direction, but not significant, or barely so, or only by one-tailed tests’. N. C. Smith (1970, p. 974) notes how replication studies are neglected:

‘The review of the literature on replication and cross-validation research has revealed that psychologists in both research “disciplines” have tended to ignore replication research. Thus, one cannot help but wonder what the impact might be if every investigator repeated the study which he believed to be his most significant contribution to the field’.

Samelson (1980, p. 623) notes in the specific context of Watson’s ‘Little Albert’ study: ‘Beyond this apparent failure of internal criticism of the data is another one that is even less debatable: the clear neglect of a cardinal rule of scientific method, that is, replication’.

As there was sufficient awareness of the problematic lack of replication research during the first crisis, one might wonder which changes during the second crisis led to a widespread recognition of the problematic nature of the lack of replication studies. I will discuss five underlying reasons: 1) the publication of studies that failed to replicate highly cited findings in the field, 2) the attention that these replication failures received due to heated discussions over the internet, 3) a more widespread understanding of the consequences of research practices that were largely normative but could potentially substantially inflate error rates of statistical tests, 4) making it more acceptable to interpret original findings as Type 1 errors, and 5) meta-scientific evidence that the rate at which studies could be replicated was surprisingly low.

### High-Profile Replication Failures

A first notable change in the second crisis is that failures to replicate have more systematically started to appear in the scientific literature, of which the Reproducibility Project: Psychology (Open Science Collaboration, 2015) has received the most attention, but single studies (Doyen et al., 2012; Ritchie et al., 2012) and multi-lab replication studies (Klein et al., 2014; Simons et al., 2014) have been equally important. Instead of general statements that findings can be difficult to replicate, there are now high-profile replication failures. These failed replications draw attention for several reasons. First of all, researchers had become increasingly frustrated by the fact that certain studies continued to be highly cited even though, through informal communication channels, the community knew very well that the findings could not be replicated. As a personal anecdote, around 2006, my PhD supervisor suggested that there should be an independent committee of respected scientists who would conduct replication studies of classic findings, especially those that the community believed might be false positives. This idea emerged when discussing a study on behavioral priming published by John Bargh and colleagues (Bargh et al., 1996). The main claim in this paper is that priming people with the concept of ‘elderly’ could slow down the speed at which they walked. It is interesting to note that, at this time, a few years before the second crisis started, it felt practically impossible for any single scientist to perform and publish a replication study with the goal to show the original finding was a Type 1 error.

And yet, early on in the second crisis, this is exactly what happened: Stephane Doyen and colleagues published a failure to replicate the elderly-priming study in 2012 (Doyen et al., 2012). I had first met Stephane Doyen at a meeting of the European Social Cognition Network in Poland in 2009, and I was impressed that someone finally publicly presented a failed replication of this classic study. It proved difficult to publish, as a specific reviewer provided negative reviews whenever they submitted the study. There are two notable aspects of the fact that the failed replication finally appeared in the public literature. The first is that the article appeared in an open access journal, PLOS One. This journal does not focus on the novelty of the results but only on how methodologically rigorous the study is. Such open access journals were the first to publish replication studies during the second crisis. Had these journals not existed, it might have been more difficult to publish replication studies. Second, Doyen and colleagues also reported a second study that identified a possible moderator. When they measured walking speed automatically with a sensor, they found no effect, but when a human experimenter measured walking speed with a stopwatch, they replicated the original effect. Including a study with this moderator adhered to the norms that existed in the field where—in line with Sidman’s (1960) ideas—failures to replicate are attributable to a lack of understanding of the conditions under which the effect emerges.^1^

If we look at how this replication study is cited, the moderator of experimenter bias is rarely, if ever, mentioned. Instead, the finding is cited as a high-profile replication failure. We might speculate that at the start of the replication crisis, psychologists were still acculturated in a system where the main cause of failed replications was thought to lie in moderators instead of Type 1 errors (Sidman, 1960). This can also be seen in the response of more senior researchers to other failed replications, such as when Stroebe and Strack (2014, p. 64) argue, ‘Thus, one reason why null findings are not very interesting is because they tell us only that a finding could not be replicated but not why this was the case. This conflict can be resolved only if researchers develop a theory that could explain the inconsistency in findings’. The idea that the original finding could be a false positive is largely absent from the first crisis, and at the start of the second crisis, some senior researchers (many of whom are responsible for editorial decisions) still had the working model of science where a new theory needs to explain discrepancies between original and replication studies. Of course, sometimes failed replications are due to moderators, sometimes they are due to Type 1 errors, and sometimes they are due to false negatives. It can be difficult to predict which of these three possibilities is at play in any replication study (Ebersole et al., 2020), but it should be possible to publish failed replications to identify Type 1 errors (Schmidt, 2009).

### Repligate

A second reason failures to replicate drew attention can be attributed to the heated discussions between original researchers and researchers who performed replication studies. Original researchers were sometimes highly defensive, and researchers on both sides would engage in ad hominem remarks. The fact that these responses were increasingly available to the scientific community through unmoderated channels such as blogs and tweets provided a level of drama that was difficult to ignore. For example, John Bargh wrote a blog post with the title ‘Nothing in their heads,’ in which he responded to the failed replication study by Doyen and colleagues. He argued that PLOS One ‘obviously does not receive the usual high scientific journal standards of peer-review scrutiny’ and that one possible reason a study can fail to replicate is ‘Incompetent or ill-informed researchers performing the replication study’. When subsequent replication studies of other scientific articles by John Bargh also failed to observe significant results, a consensus emerged that failures to replicate were not attributable to incompetent replication researchers. For a few years, there was a substantial amount of interpersonal conflict as the community struggled to learn how to deal with the fact that peers could now publish failed replications of your work.

These conflicts reached a peak (then referred to as ‘repligate’) after the publication of a special issue of the journal Social Psychology contained a set of replication studies that were published as Registered Reports (Nosek & Lakens, 2014). Registered Reports are peer-reviewed and accepted for publication before the data are collected and will be published regardless of the statistical significance of the results, as long as authors follow their preregistered plan (Chambers, 2013). Researchers who had published the original studies that were replicated contacted the editor in chief of Social Psychology, arguing they had the right to respond to failed replications of their work. The fact that authors felt entitled to respond to replication studies of their work is indicative of how psychological scientists had to learn how to respond to published replication studies. A decade later, it has become much rarer for authors to submit a commentary article in response to a replication of their work. Discussions following the publication of the special issue got quite heated, especially regarding the failure to replicate studies about the link between cleanliness and moral judgments published by Simone Schnall. She stated, ‘I feel like a criminal suspect who has no right to a defense and there is no way to win’ (Bohannon, 2014). Discussions in blogs and on social media led to personal attacks (Holcombe, 2022), most notably by the psychologist Daniel Gilbert, who criticized his peers involved in replication research as ‘shameless little bullies’ and the ‘replication police’.

During the first crisis, failed replications—if they were published at all—did not lead to contentious disagreements. The few replications that were performed drew less attention and did not leave a lasting impression on the field. For example, consider the evaluation of Schachter’s cognition-arousal theory of emotion by Reisenzein (1983). This systematic review contains a description of several failed replications and methodological confounds. In this case, the original author also strongly criticized some of these failed replications, for example, by remarking, ‘We find ourselves both bemused and perplexed by their strategy of replication’, and report numerous differences between their studies and failed replications that they believe explain why different results were observed (Schachter & Singer, 1979).^2^ A noticeable difference is that failed replications in the first crisis are rarely as close as possible to the original studies, which makes it easier for original authors to attribute the failure to moderators. Furthermore, because communication between authors mainly occurs through published and peer-reviewed journal articles, there seems to have been less drama. During the second crisis, the ability of scientists to communicate quickly and directly through social media and blogs generated more attention for discussions about how the field should perform, publish, and respond to replication studies.

### Awareness of Type 1 Error Inflation

A third reason failed replications received more attention during the second crisis was the awareness that it is possible to report multiple statistically significant tests of a hypothesis, even when there is no true effect. Daryl Bem’s article, in which he claimed to have found empirical support for precognition, contained nine studies (Bem, 2011). In these studies, participants performed traditional tasks in experimental psychology, but with one twist: instead of presenting the stimulus that participants needed to respond to before they needed to respond to the stimulus, participants needed to respond to a stimulus that would be presented in the future. For example, instead of indicating if a picture appeared on the left or right side of the screen, participants had to indicate where a picture would appear. Performance statistically better than the guessing average was taken to indicate that people had the ability to predict the future. This claim does not only contradict common sense but also all working models of physics. And yet, he was able to present nine studies which, under all normal circumstances, should be extremely convincing evidence of a true effect.

Instead of increasing their belief in the precognitive abilities of human beings, most psychologists responded by questioning the research practices that Bem had used to test hypotheses. An impactful paper by Simmons et al. (2011) convincingly explained how certain practices could substantially inflate the Type 1 error rate of significance tests. These techniques, later referred to as ‘p-hacking’, increase the probability that a claim is a false positive, especially when multiple of these techniques are combined. Psychologists rediscovered earlier articles in the scientific literature that discussed how low power, inflated alpha levels, and publication bias could lead to a scientific literature filled with false positives (Neher, 1967; Wacholder et al., 2004). The provocatively titled article by John Ioannidis, ‘Why most published research findings are false,’ drove home the realization that a combination of factors could lead to a very unreliable scientific literature (Ioannidis, 2005). Although the pessimistic scenario sketched by Ioannidis is unlikely to hold for science overall, some research lines are now strongly suspected to mainly consist of false positives.

For example, Carter and McCullough (2014) re-analyzed a published meta-analysis of 198 independent tests of the ego-depletion hypothesis and identified strong bias in the set of studies. Estimates of the unbiased effect size estimate raised the possibility there was no effect at all. In 2016 a preregistered replication study with 2141 participants found a non-significant ego-depletion effect that was basically zero (Hagger et al., 2016). In 2021 a preregistered replication study with 3531 participants, performed by the original authors of ego-depletion studies, also found a non-significant effect very close to zero (Vohs et al., 2021). This research line empirically demonstrated the extreme consequences publication bias can have on the scientific record, where there can be 198 statistical tests indicating there is an effect, with unbiased replication studies suggesting there was no effect all along. Such findings strongly contributed to the sense of a replication crisis, as researchers were increasingly uncertain about whether the research they built on was reliable.

### Attributing Failed Replications to False Positives

The fourth reason replication studies received more attention was that it became easier to attribute a failed replication to a false positive finding in the original study. Because the idea that people have the ability to predict the future is theoretically so implausible (leading one reviewer of Daryl Bem’s paper to recommend rejecting the manuscript because it was impossible to theoretically explain the results), no one required researchers who attempted to replicate Bem’s studies to develop an alternative theory to explain away the discrepancies (Galak et al., 2012; Kekecs et al., 2023; Ritchie et al., 2012). While such alternative explanations were still expected to publish failed replications of behavioral priming effects (Doyen et al., 2012), psychologists increasingly accepted the fact that sometimes there is nothing to explain. This subsequently made it easier to publish direct replication studies, as there was no longer an implicit requirement to explain away discrepancies between the original and replication study. Registered Reports offered another route to publish well-designed replication studies while preventing reviewers from critiquing a study after the results are known (Nosek & Lakens, 2014).

### Empirical Metascience

A final difference in the second crisis is the emergence of the field of metascience, which empirically investigates the scientific process itself (Faust & Meehl, 1992). Empirically quantifying the extent to which scientific findings replicate is a challenging problem, as was already acknowledged in the Reproducibility Project: Psychology (2015, pp. aac4716–7), where replications of 100 studies were performed:

‘After this intensive effort to reproduce a sample of published psychological findings, how many of the effects have we established are true? Zero. And how many of the effects have we established are false? Zero. Is this a limitation of the project design? No. It is the reality of doing science, even if it is not appreciated in daily practice. Humans desire certainty, and science infrequently provides it. As much as we might wish it to be otherwise, a single study almost never provides definitive resolution for or against an effect and its explanation’.

Even though the extent of the replicability problem remains difficult to quantify, the second crisis provided empirical evidence of the fact that failures to replicate are non-negligible, at least in certain research areas. Of course, what constitutes ‘non-negligeable’ is a subjective judgment, and which areas suffer from low replicability remain uncertain unless large replication projects are performed. I believe that most researchers in psychology expected that if they attempted to follow the procedures described in a previous study and collected sufficient data to have high power for effect sizes reported in the literature, a greater percentage of studies should yield a significant *p*-value than the 36% observed in the Reproducibility Project.

Empirical research into replication success has revealed that it is incredibly difficult to establish whether effects are replicable. It requires a careful and systematic examination of differences between original and replication studies. Researchers have made the case on theoretical grounds that the only way to know if an effect will replicate is to replicate it (Miller & Schwarz, 2011). A recent multi-lab replication project, Many Labs 5, has provided empirical support for this idea (Ebersole et al., 2020). Re-examining ten studies that failed to replicate in the Reproducibility Project, these novel replication studies revealed that after an initial failed replication study, anything can happen. In two of the replication studies, changes to the procedure that were suggested by the original authors led to a successful replication, while the version of the procedure used in the Reproducibility Project yielded no significant effect. These studies demonstrate that sometimes failed replications are due to methodological differences between original and replication studies. However, both these studies also yielded extremely small effect size estimates, which leads to the question of which effects in replication studies are large enough to matter (Simonsohn, 2015). In six other studies, the reasons raised by the original authors for the failure to replicate did not matter, and the replication studies once again failed to yield statistically significant results; sometimes their suggestions for improvement led to results that were even less in line with their original results. One study yielded non-significant results in all replication studies, but effect size estimates were very similar, raising the possibility of Type 2 errors, as there might be a real but very small effect. A final study yielded more variable results but also hinted at a very small true effect size. Given resource restraints, most researchers would consider the observed effects in these large replication studies practically insignificant, even if they were statistically significant (Lakens et al., 2018). The empirical results have illustrated how much work the field of psychology needs to do to establish and implement methodological procedures to determine if effects have replicated or not (Anderson & Maxwell, 2016; Uygun-Tunç & Tunç, 2023). Altogether, it has become clear that successfully replicating a finding in the published literature is substantially more challenging than many researchers in psychology expected.

## By whom and how should replications be performed?

As the realization increased that replication studies were important to perform and publish, but establishing whether a finding could be replicated also required substantial effort, the question arose of which studies should be replicated. Not all replications are equally useful. First of all, there is little use in replicating a badly designed study, as was already noted by Rosenthal (1966, p. 327) during the first crisis: ‘A very badly done experiment profits us little, upon even many replications’, and noted, for example, by Pittelkow and colleagues (2021, p. 212) during the second crisis.

‘Replication studies for which the original methodology may not have been optimal […] may not have as much merit as replication studies for which the methodology […] was comparatively high. Such considerations are important in times where limited funding means replicating every single study may not be feasible’.

Researchers in general believe studies should be replicated when they have had some form of impact and when the result has remaining uncertainty, but it remains an open question how impact and uncertainty should be operationalized or weighted (Isager et al., 2023).

A more contentious question is whether we can perform a similar enough replication study in a field such as psychology. During both crises researchers acknowledge it is impossible to perform the same study twice. ‘But to avoid the not very helpful conclusion that there can be no replication in the behavioral sciences, we can speak of relative replications. We can order experiments on how close they are to each other in terms of subjects, experimenters, tasks, and situations’ (Rosenthal, 1966, p. 321). This issue became much more pertinent when researchers actually started to perform direct replications during the second crisis and remains an ongoing topic of discussion. A more worked-out proposal comes from Lebel and colleagues (2017, p. 255) during the second crisis, who note, ‘Replications lie on an ordered continuum of methodological similarity to an original study, ranging from highly similar to highly dissimilar’. They suggest specifying different aspects of the design (e.g., the operationalization of the independent and dependent variable, the stimuli, the procedure, the context, etc.) and listing the similarity to the original study for each aspect. During both crises, researchers have pointed out there is a role for both conceptual (‘constructive’) replication studies and direct (‘operational’) replication studies (Crandall & Sherman, 2016; Lykken, 1968; Zwaan et al., 2018), even though there has also been criticism of the idea that we should perform more direct replication studies during both crises (Hunt, 1975; Stroebe & Strack, 2014).

Gergen (1973) questioned whether most (if not all) effects in psychology are not stable across time and context and, therefore, if replication was a reasonable expectation in psychology. Gergen’s work introduced an important theme in the first crisis in social psychology, namely the question of whether findings in psychology could be expected to generalize. Gergen writes, ‘It is the purpose of this paper to argue that social psychology is primarily an historical inquiry. Unlike the natural sciences, it deals with facts that are largely nonrepeatable and which fluctuate markedly over time’ (1973, p. 310).^3^ Gergen does not necessarily dismiss the possibility that some effects turn out to be quite durable, but whether effects are stable over time is anything but certain: ‘We must think, then, in terms of a continuum of historical durability. with phenomena highly susceptible to historical influence at one extreme and the more stable processes at the other’. This work fueled interest in the social constructionist movement in psychology (Gergen, 1985).

Despite the impact Gergen’s views had on the first crisis, there were also critics of his arguments (e.g., Manis, 1975). The social constructionist perspective has largely (although not completely) disappeared as a topic of discussion during the second crisis. This does not mean that constructivist approaches have disappeared, especially among qualitative researchers. But it is rare to see researchers take the notion that replicability should not be an aim in psychological science seriously in response to failed replications. The question of whether failures to replicate are due to contextual factors continues to be a topic of discussion (Pettigrew, 2018; Uygun-Tunç & Tunç, 2023; Van Bavel et al., 2016). An intriguing change during the second crisis is a more pragmatic approach to the question of whether failures to replicate should be attributed to differences in the context. Instead of putting the burden of proof that there are no contextual moderators on the researcher who performs a replication study, the original authors are held accountable for any failure to specify the context to which their finding is expected to generalize. By requiring researchers to specify a ‘Constraints on Generality statement’ (Simons et al., 2014), it now becomes the responsibility of the original researcher to state which contextual moderators they believe would make effects they have observed disappear. Replication studies can now sample intentionally from within or beyond the specified constraints on generality to perform informative tests of the replicability and generalizability of findings. Furthermore, although failures to replicate have perhaps received most of the attention, we should not forget that many studies have been successfully replicated, providing empirical support for the idea that it is possible in practice to perform sufficiently close replication studies that demonstrate stable effect sizes.

## A Lack of Incentives for Replication Studies

During both crises researchers point out that the incentive structure does not reward replication studies. ‘Some journals explicitly state that they do not accept replication studies in principle, while others implicitly follow a similar policy; it is not surprising that few are ever published’. Little changed, much to the dismay of many scientists, as illustrated by the public outcry when, at the start of the second crisis, failed replications of Bem’s precognition study were desk-rejected by the editor of JPSP, Eliot Smith, who stated, ‘This journal does not publish replication studies, whether successful or unsuccessful’, and, ‘We don’t want to be the Journal of Bem Replication’. Some researchers proposed novel publication formats to solve the problem. For example, Ahlgren discusses a ‘hypothetical Journal of Replicated Studies in Psychology’ which publishes ‘only those studies that had been successfully replicated (whether the null hypothesis is rejected or not)’, while ‘in the back of the Journal would be listed abstracts of studies that have been provisionally accepted for publication, contingent on successful replication’ and ‘also in the back might be an edifying (and sobering) table of titles of previously listed abstracts, marked ‘pending replication’, ‘supported by replication’, or ‘confounded by replication’. Relatedly, Rosenthal (1966, p. 323) states that ‘in order to benefit properly from replications actually carried out, it is essential that these be routinely published, even if only as brief notes with fuller reports available from the experimenter, from a university library, or from the American Documentation Institute’. Similar calls to publish brief summaries of replication studies have been made in the second crisis:

‘Replications could be published in a special section of each journal, much like the sections that are currently used for making editorial statements. If journals still appear in print, the print version of a journal issue could contain short abstracts of new replications. The complete manuscripts of replications could be published as supplementary online materials, which become instantly available upon accessing the original report’.

None of these recommendations have been implemented.

However, an important change during the second crisis is that standalone replication studies have become easier to publish. In addition to new open access journals, the development of the novel Registered Report publication format provided an additional outlet for replication studies (Nosek & Lakens, 2014). An increasing number of journals started to explicitly invite high-quality replication studies. There are remaining issues with the amount of prestige researchers get when they publish a replication study compared to when they publish a novel finding. This raises the question of who should complete these replication studies, which poses something of a social dilemma: It is in the interest of science that replication studies are performed, but it is in the interest of the individual researcher to perform novel research.

Rosenthal suggests that researchers can divide replication studies among themselves and writes:

‘Who, then, on any large scale will provide us with the necessary replications? McGuigan’s data and Woods’ suggest that there are now enough experiments carried out and reported by multiple authors for there to be no hardships in subdividing these studies into as many complete replicates as there are investigators. The total investment of time would not be increased, but the generality of the results would be’ (Rosenthal, 1966, p. 323).

A similar idea to distribute replications across researchers is proposed during the second crisis:

‘However, a potential barrier to independent, prepublication replication attempts is that many researchers have a tough time finding other labs to conduct such attempts. StudySwap can be used to find an independent research team to conduct a replication attempt of a not-yet-published study’ (Chartier et al., 2018, p. 575).

Another solution to increase the number of direct replication studies that are performed is to let students perform replication studies. Schlosberg already noted (1951, p. 177):

‘Why don’t psychologists repeat fundamental experiments, as do physicists? This question has popped up in several informal discussions in which I happened to participate. […] Such repeat experiments are very satisfactory for laboratory courses, honors candidates, and even MA theses. They furnish excellent training’.

The same point is made by Frank and Saxe (2012, p. 600) during the second crisis:

‘Replication is held as the gold standard for ensuring the reliability of published scientific literature. But conducting direct replications is expensive, time-consuming, and unrewarded under current publication practices. So who will do them? The authors argue that students in laboratory classes should replicate recent findings as part of their training in experimental methods’.

However, whether we can expect students to replicate experiments has been questioned. Sidman (1960, p. 109) remarks, ‘Failures to replicate must be evaluated in terms of the background and training of the experimenter, even though research in other areas has gained him a respected reputation’.

This sentiment must be true to some extent, but the available meta-scientific evidence suggests that experts often have an equally difficult time replicating their own work. For example, Baumeister responded to failures to replicate his work on ego-depletion by stating:

‘I submit that some experimenters are incompetent. In the past their careers would have stalled and failed. But today, a broadly incompetent experimenter can amass a series of impressive publications simply by failing to replicate other work and thereby publishing a series of papers that will achieve little beyond undermining our field’s ability to claim that it has accomplished anything’.

Yet when his close collaborator led a team of experts in a replication project of the ego-depletion literature, they also failed to find significant ego-depletion effects despite very large sample sizes (Vohs et al., 2021). Too few of the researchers who responded defensively when peers failed to replicate their work have responded by publishing a Registered Report in which they successfully replicate their own work, but there have been some inspirational examples (e.g., Giessner & Schubert, 2019; Luttrell et al., 2017). Adversarial collaborations, where original researchers and replicators skeptical of the original finding work together, offer one way to perform high-quality, informative replication studies (Mellers et al., 2001).

## Discussion

It is not surprising that one of the main successes from the second crisis—increasing the awareness of the difficulties of replicating published findings—was the result of several large-scale projects. The Reproducibility Project, a five-year-long collaborative research effort among 270 researchers, is perhaps the most widely known, but a number of similar large-scale projects provided empirical evidence that it is often difficult to replicate published findings, even with the help of original researchers (Ebersole et al., 2020; Vohs et al., 2021). The amount of work that went into these projects is well beyond the resources any individual scientist has at their disposal. Collectively, these projects seem to have motivated researchers in psychology to improve research practices related to replication studies. This includes creating journal policies to publish replication studies, examining how to best analyze replication studies, and performing replication studies. It seems plausible that other challenges, such as improving measurement or increasing the relevance and applicability of psychological research, will also require a long-term coordinated research effort to finally instigate change. Effective coordination in science relies on shared goals, open communication, the integration of interdependent contributions by specialists, and management that takes responsibility for quality assurance (Rasti et al., 2025b; Rasti et al., 2025a).

Alternatively, solutions can come from effective governance institutions that induce rule compliance. An example is the adoption of clinical trial registries based on rules that have been developed by government organizations, funders, and journals. Another example is the work of cOAlition S, where a large number of science funders cooperate to make research publications open access. Although there is often resistance to rules that are implemented in a top-down manner, when goals and values are clearly communicated, policies are based on a dialogue between all stakeholders, rules are enforced fairly, and there is sufficient support, this approach has the potential to lead to meaningful change. An example from the first crisis is how ethical review procedures were developed after extensively consulting stakeholders, including academic psychologists. To resolve other challenges, one could imagine that funders, journals, and professional organizations collaborate on policies to decide which studies should be replicated by the community (Isager et al., 2023), consensus meetings where scientific fields come together to improve and standardize the measures they use, or journal editors who will request the adoption of better methods in author guidelines.

## Footnotes

It is worth noting that the sample size in this second study is very small, and the claim that a moderator exists is based on the fact that one simple effect was significant, while the other simple effect was not significant, instead of a direct test of the interaction effect.

As an interesting aside, Schachter and Singer also complain how “in these days of ethical guidelines and human subjects committees […] it is unlikely that anyone will do experiments such as ours […] for quite a while, if ever again”, illustrating the salience of the recent changes in research ethics.

Whether there is actually more variability in the social sciences than in the physical sciences has been contested (Hedges, 1987).
